# Association between motor competence and Functional Movement Screen scores

**DOI:** 10.7717/peerj.7270

**Published:** 2019-08-08

**Authors:** Bruno Silva, Luis Paulo Rodrigues, Filipe Manuel Clemente, José M. Cancela, Pedro Bezerra

**Affiliations:** 1 Escola Superior de Desporto e Lazer de Melgaço, Instituto Politécnico de Viana do Castelo, Melgaço, Portugal; 2 Research Center in Sports Sciences, Health and Human Development (CIDESD), Vila Real, Portugal; 3 Faculty of Education and Sport Sciences, Universidad de Vigo, Pontevedra, Spain; 4 Instituto de Telecomunicações, Covilhã, Portugal

**Keywords:** Motor development, Physical function, Young adults, FMS™, Human movement

## Abstract

**Background:**

Motor competence (MC) is generally used to describe a person’s proficiency in a variety of motor skills and is the basis for one’s performance in sports and recreational activities. Functional Movement Screen (FMS^TM^) is one of the most used screening systems to provide interpretable measure of movement quality. Both FMS^TM^ and MC constructs share three components: locomotor, manipulative and stabilizing movements. In the present study, it was hypothesized that MC scores can explain FMS^TM^ variables. It was also predicted that better MC leads to better functional movement patterns in young adults.

**Methods:**

A sample of 92 young adults (73.9% men) with a mean age of 21.2 years participated in this study. All participants were evaluated on anthropometric measurements, dual x-ray absorptiometry; FMS^TM^ and MC.

**Results:**

Men showed better MC scores and fat mass composition than women. Regarding specific tests, women scored higher in the FMS^TM^active straight leg raise test, whereas men performed better in the FMS^TM^ trunk stability push-up (TSP) test. Manipulative tasks and construct presents’ significant and positive associations with FMS^TM^ composite score (*r* ≥ 0.303). The significant negative correlation were more related to FMS^TM^ TSP and MC shuttle run and FMS^TM^ in-line lunge and MC manipulative. The FMS^TM^ TSP presents significant associations with all MC constructs and tasks. Meanwhile, the FMS^TM^ composite score is associated with all components of MC Stability (*p* < 0.05). In young adults, and independent of gender, the FMS^TM^explains fundamental movements based on motor control according to the stability construct. Moreover, the FMS^TM^ TSP is associated with better performance in the all MC constructs and MC tasks. The FMS^TM^, on its own, is linked to objective MC stability measures.

## Introduction

Movement competency is an integral component of physical literacy (Whitehead, 2010) and is thought to be a fundamental aspect of childhood development (Ahnert, Schneider & Bös, 2011; Stodden et al., 2008). In the initial phases of motor development, children’s motor competence (MC) involves the mastery of fundamental motor skills that are the foundation of the mastery of specialized motor skills (Luz et al., 2016).

Assessing a child’s motor repertoire of movements and their ability to perform these movements may provide insight into the relationship between the development of the nervous system and the overall developmental process (Gallahue, Ozmun & Goodway, 2012). Motor competence, as it relates to the development and performance of human movement (Stodden et al., 2008), is generally used to describe a person’s proficiency in a wide variety actions and motor skills (gross and/or fine) (Fransen et al., 2014). Motor competence also serves as a basis for their ability to perform sports and recreational activities. Motor competence depends on the optimal development of fundamental motor skills, comprising locomotor, stability, and manipulative tasks (Gallahue, Ozmun & Goodway, 2012; Luz et al., 2016). Motor competence has been found to correlate positively with physical activity and physical fitness and negatively with weight status among developing children (Robinson et al., 2015). Interestingly, longitudinal studies have shown that MC is a strong predictor of physical activity (Lopes et al., 2011) and physical fitness status (Rodrigues, Stodden & Lopes, 2016).

There are several standardized and non-standardized protocols that can be used to assess MC (Bardid et al., 2018; Okely, Booth & Chey, 2004). However, MC is a complex concept that assesses an individual’s proficiency in executing motor skills, making it difficult to derive a universal measure of MC. The absence of a standard measure has led researchers to consider the purpose of assessing population characteristics and the range of practical aspects that determine which instrument should be used in any given case (Bardid et al., 2018). Additionally, the theoretical construct is not always reflected in the majority of available assessments (Luz et al., 2016). The recent motor competence assessment (MCA) battery can solve this problem with an easy and reliable assessment of the three major latent variables of MC (Luz et al., 2016).

Movement proficiency falls under the spectrum of MC because it reflects the underlying processes of movement, such as coordination, control, and movement quality (Gabbard, 2008). Functional movement is another indicator of movement proficiency (O’Brien et al., 2017).

Concerning the daily practice, the Functional Movement Screen (FMS™) is one of the most commonly used screening systems; it provides a clinically interpretable measure of movement quality (Kraus et al., 2014; Marques et al., 2017). The FMS™ was designed to assess the functional movement patterns of an individual because of the importance of inspecting and understanding common fundamental aspects of the human movement (Cook, 2011; Cook et al., 2014b). The FMS™ is composed of a set of seven tests (Cook, Burton & Hoogenboom, 2006; Kraus et al., 2014), creating a functional movement baseline, which work together to create a functional movement baseline, which allows for the rating and raking of movement (Cook et al., 2014b). Each test is characterized by a specific movement which provides observable basic locomotor, manipulative, and stabilizing movements, all of which require the participant to perform common fundamental movement patterns (Cook et al., 2014b).

The MCA battery objectively monitors motor development and is representative of MC, making it easy to assess quantitative information (Luz et al., 2016). The FMS™ is used to screen individual movement inefficiencies in order to assess an individual’s dynamic and functional capacities and their readiness to return to physical activity after rehabilitating from an injury or surgery (Cook et al., 2014b; Silva et al., 2017).

The Functional Movement Screen and MC constructs share three components: locomotor, manipulative and stabilizing movements. However, few studies have investigated the possible relationship between FMS™ scores and MC levels. By studying young adults, the data may provide new insight into the movement proficiency barrier which emerges during middle childhood and adolescence and becomes more clearly defined during young adulthood (Stodden, Langendorfer & Roberton, 2009). This analysis may help researchers and health and sports training professionals make better-informed decisions by objectively measuring fundamental motor task performance.

Following this, the aims of this study are (1) to investigate the associations between MC and FMS™ scores; (2) to analyze the correlation between MC and FMS™ manipulative, locomotor, and stability tasks; (3) to understand whether MC scores can explain FMS™ scores; and (4) to observe differences between male and female participants. We hypothesize that MC scores can explain FMS™ variables; we also predict that better MC leads to better functional movement patterns in young adults.

**Table 1:** Anthropometric sample description (mean and 95% CI).

**Variable**	**Men (*n* = 68)**	**Women (*n* = 24)**	***p*-value**	**Effect size**
Age (years old)	20.1 [19.5–20.8]	19.70 [19.1–20.3]	0.088	Small effect
Height (cm)	176.6 [174.5–178.7]^*^	162.6 [160.0–162.3]	0.000	Large effect
Weight (kg)	74.3 [71.2–77.3]^*^	58.1 [54.6–61.9]	0.000	Large effect
Body fat (%)	22.4 [20.5–24.3]	33.0 [30.3–35.8]^*^	0.000	Large effect
Bone mineral density (g/cm^2^)	1.3 [1.2–1.3]^*^	1.2 [1.1–1.2]	0.000	Medium effect
Total Lean (g)	13182.9 [12698.6–13667.3]^*^	9224.3 [8711.6–9737.0]	0.000	Large effect

**Notes.**

cm centimeters kg kilograms % percent g/cm^2^ grams per square centimeters g grams

* *p* < 0.05.

## Material & Methods

### Participants

A sample of 92 young adults (73.9% male) with a mean age of 21.2 years (Table 1) participated in this study. The participants were volunteers, consisting of 68 male (22.3 years; 69.9 kg; 172.7 centimeters) and 24 female (20.7 years; 65.9 kg; 170.9 centimeters), all of whom were students in a Faculty of Sports Sciences undergraduate course. Participants had no motor, cognitive, or health impairments that could affect their performance on the tests. Participants were informed of the study design and of the potential risks and benefits of their participation. After being briefed, participants signed a free informed consent in accordance with the ethical standards for the study in humans as suggested by the Declaration of Helsinki. The study was approved by the Board and the Scientific Committee of the School of Sports and Leisure of the Polytechnic Institute of Viana do Castelo (CTC-ESDL-CE002-2017).

### Procedures

All participants were evaluated by the FMS™ and the MCA and then had their anthropometrics measures taken. The data were collected from October to November, during the first month of the academic year. All tests were conducted at the biomechanics laboratory of the School of Sports Science.

Firstly, all subjects answered a socio-demographic questionnaire and gave their informed consent. The assessments were made during the morning period in groups of 20 participants at an average temperature of 26° Celsius and relative humidity of 18%. The tests were conducted in the following sequence: (1) anthropometric, (2) dual X-ray absorptiometry, (3) FMS™, and (4) MC. Participants received clear instructions of the procedures for the FMS™ and MC assessment and were provided with a demonstration performed by a proficient model.

#### Anthropometrics

The body weight of each participant was measured on a scale (SECA 760, Germany) to the nearest 0.5 kg, and participants’ heights were measured to the nearest 0.1 cm using a portable stadiometer (SECA 217; SECA, Hamburg, Germany). During this evaluation, the participant wore light clothing and stood barefoot, with their head oriented according to the Frankfurt plane. Body composition was measured using a General Electric Hologic Discovery scanner (Hologic Inc., Waltham, MA, USA). Dual-energy X-ray absorptiometry (DXA) was used by a certified and experimented DXA operator according to the manufacturer’s specifications. As per (Hart et al., 2015; Hart et al., 2015), the DXA operator assisted the participant to (1) straighten their head, neck, and torso parallel to the long axis of the scan bed, (2) position their shoulders and pelvis perpendicular to the long axis of the scan bed;, (3) place both arms in pronation by their side, (4) place their legs at shoulder width with a 45° internal rotation, and (5) fixate their feet together using strapping tape to minimize incidental movement and for the participant’s comfort.

Percentage of total body fat, bone mineral density and total lean mass were considered for analysis. DXA provides information on three factors of body composition, according to the terminology: “fat mass”, “lean mass” (or “fat-free soft tissue”), and “bone mineral content.”

#### Functional movement screen

The FMS™ was applied according to the battery developed by Cook et al. (2014a) and Cook et al. (2014b). This screening simplifies the assessment of fundamental movement patterns (Cook et al., 2014a; Cook et al., 2014b) according to seven movements—deep squat (DS), hurdle step (HS), in-line lunge (ILL), shoulder mobility (SM), active straight-leg raise (ASLR), trunk stability push-up (TSP), and rotary stability (RS)—and three clearing examinations. The clearing examinations (shoulder clearing test, spinal extension clearing test, and spinal flexion clearing test) were not scored but were performed to determine whether the participant was able to perform the assessments.

Three attempts of each pattern were completed, and the best repetition was scored on a scale of 0 to 3 as follows:: 0 = pain reported anywhere in the body; 1 = unable to complete the movement pattern or unable to assume the position to perform the movement, 2 = able to complete the movement but must compensate in some way to perform the fundamental movement, 3 = able to perform the movement correctly without any compensation, complying with standard movement expectations associated with each test (Cook, Burton & Hoogenboom, 2006).

A certified FMS™ specialist with four years of experience conducted the tests according to the standard protocol (Cook et al., 2014a; Cook et al., 2014b) with an official FMS™ test kit. Approximately 10 s of rest was provided between trials and one minute of rest was allowed between tests. In all tests except for the DS and TSP, each side of the body was assessed unilaterally, with the best scores for each of the seven tests registered for analysis and used to calculate a composite score. The composite FMS™ score was derived by summing the scores for individual tests. Nevertheless, it is necessary to consider the unilateral assessment. For example, an individual who received a score of 3 for the HS on the left leg and score 2 on the right leg received a final score of 2 for the HS. Each participant could achieve maximum of 21 points. The reliability of these assessment protocols has been established with moderate to excellent levels of agreement in trained raters (Minick et al., 2010; Onate et al., 2012).

#### Motor competence assessment

Motor Competence was evaluated with the MCA battery developed by Luz et al. (2016). An experienced and specialized researcher conducted the assessment, which were composed of two tests for each MC category: stability (lateral jumps (LJ) and shifting platforms (SP)), locomotor (shuttle run (SHR) and standing long jump (SLJ)), and manipulative (throwing velocity (TV) and kicking velocity (KV)).

**Figure 1:**
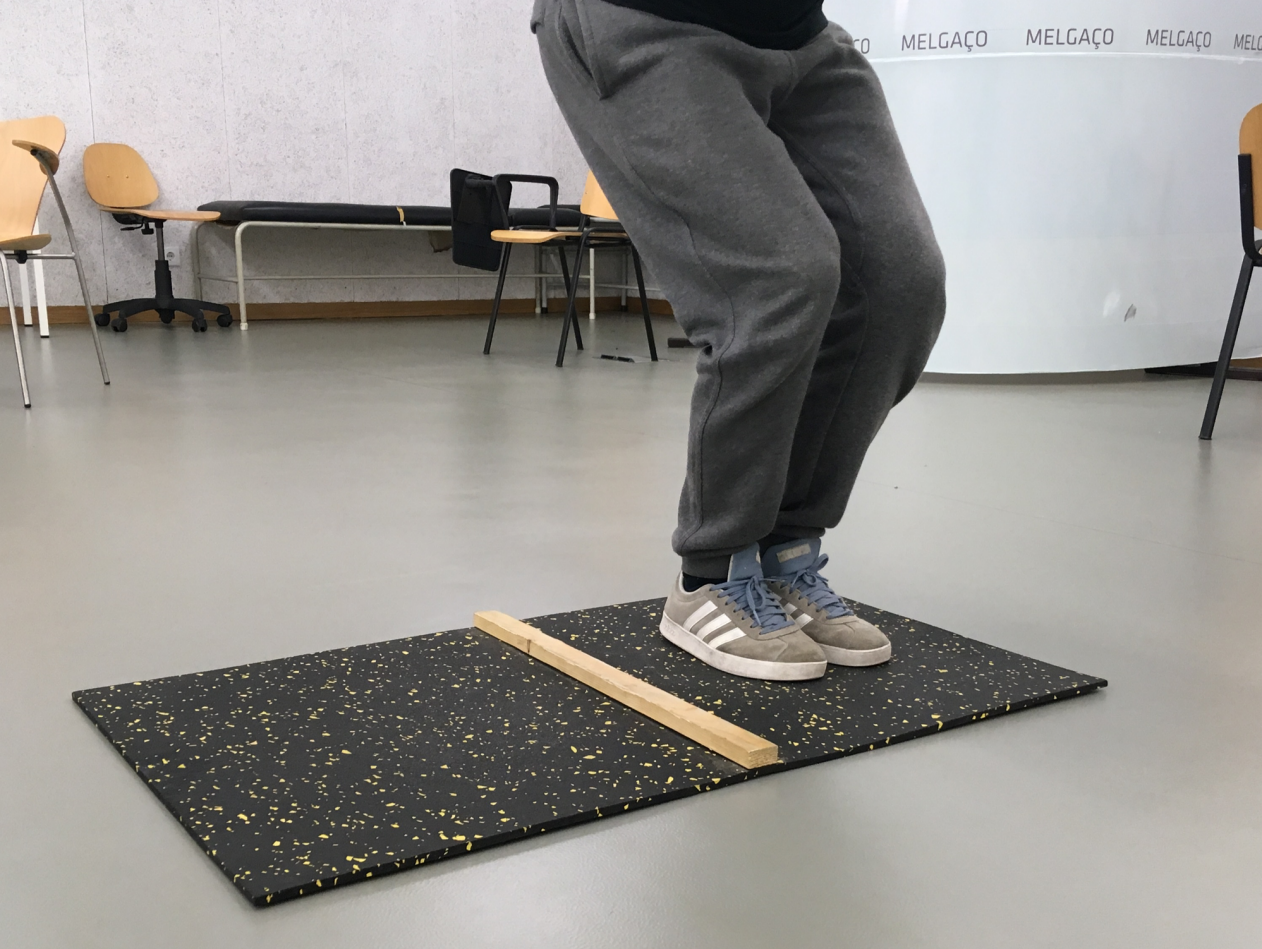
Motor competence—lateral jump test.

The **LJ tests** (Fig. 1) consisted of jumping sideways as fast as possible for 15 s. During testing, participants jumped with their feet together over a small wooden beam (60 cm length × 4 cm height × 2 cm width) located in the middle of a rectangular surface (100 cm length × 60 cm width). Each jump made without touching the outside of the rectangle and without stepping on the wooden beam was awarded one point, and the best score was recorded.

**Figure 2:**
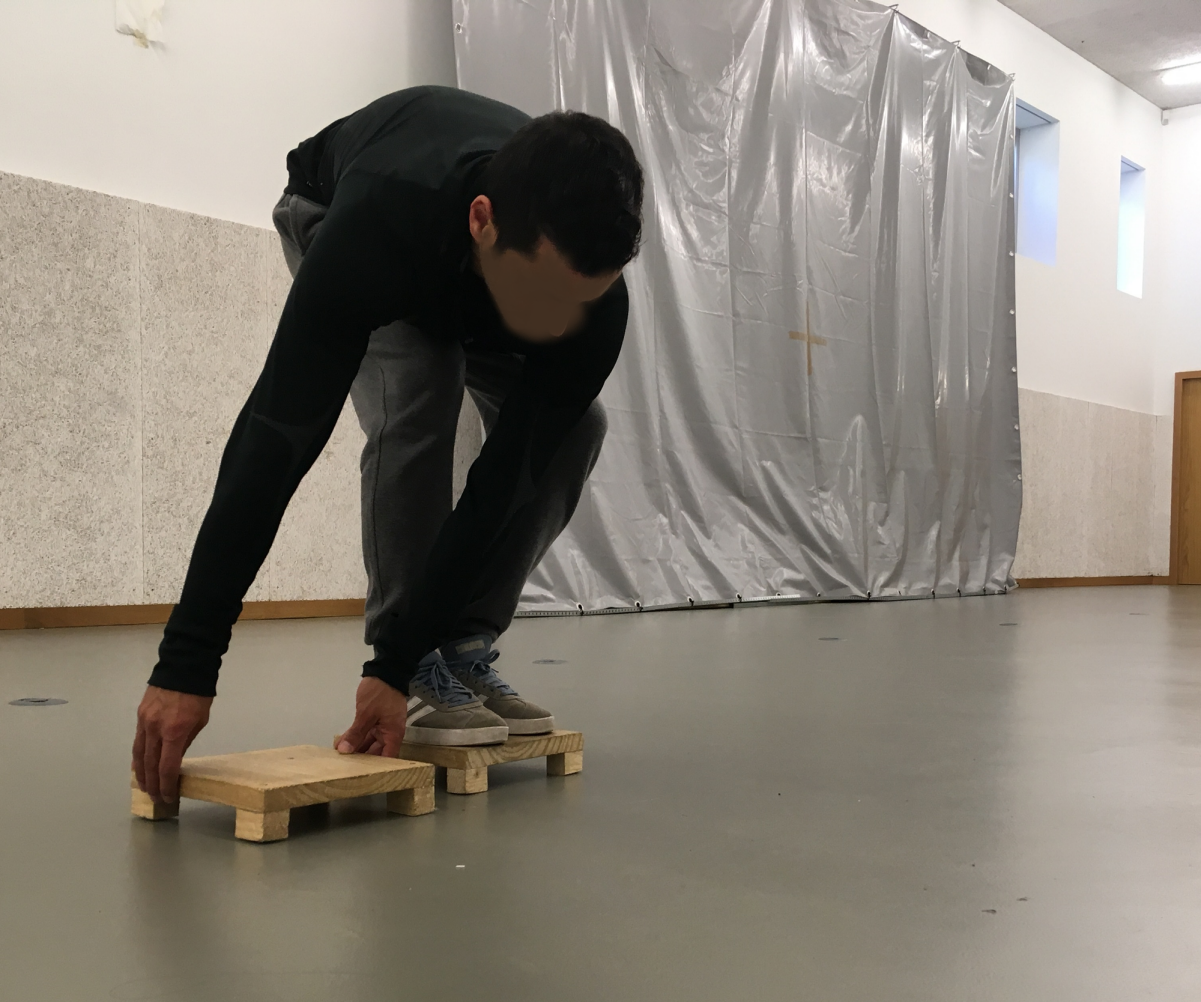
Motor competence—shifting platforms test.

The **SP** **test** (Fig. 2) required subjects to move sideways using two wooden platforms (25 cm × 25 cm × 2 cm) for 20 s. Each successful transfer from one platform to the other was scored. One point was achieved for moving the platform, and another point was awarded for moving onto the platform, with each complete successful transfer giving the participant two points. Participants completed two trials and the best score was recorded.

**Figure 3:**
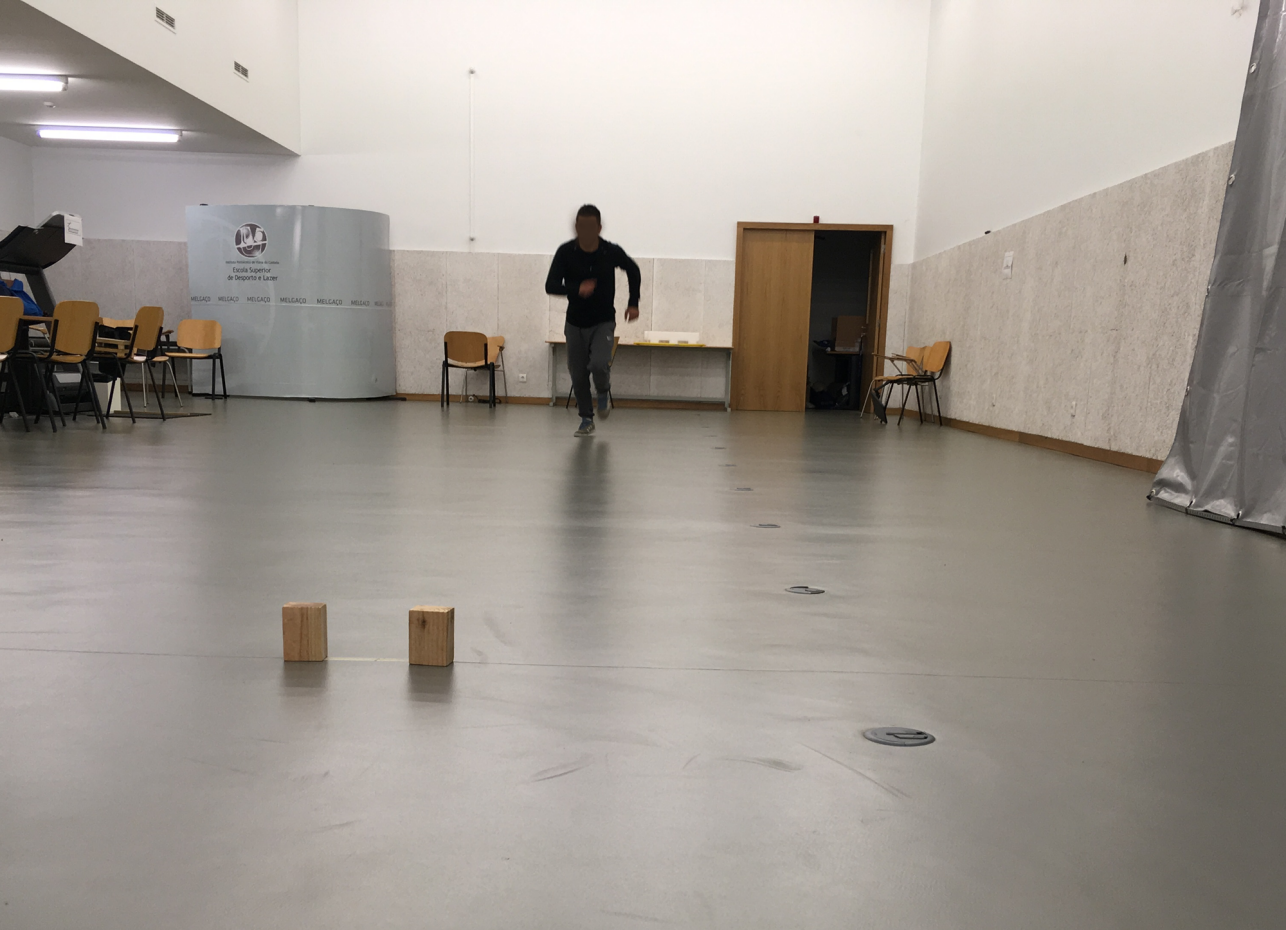
Motor competence—10-meters shuttle run test.

The **SHR** **test** (Fig. 3) required participants to run a distance of 4 × 10 m at a maximal speed between the starting and finish lines. The test began at the starting line after an acoustic starting sound was made. Then, participants ran to the finish line, picked up a block of wood, ran back and placed the block beyond the starting line. Without stopping, subjects ran back to the finish line to retrieve a second block and carry it back across the starting line to finish the test. The best time of the two trials was recorded.

**Figure 4:**
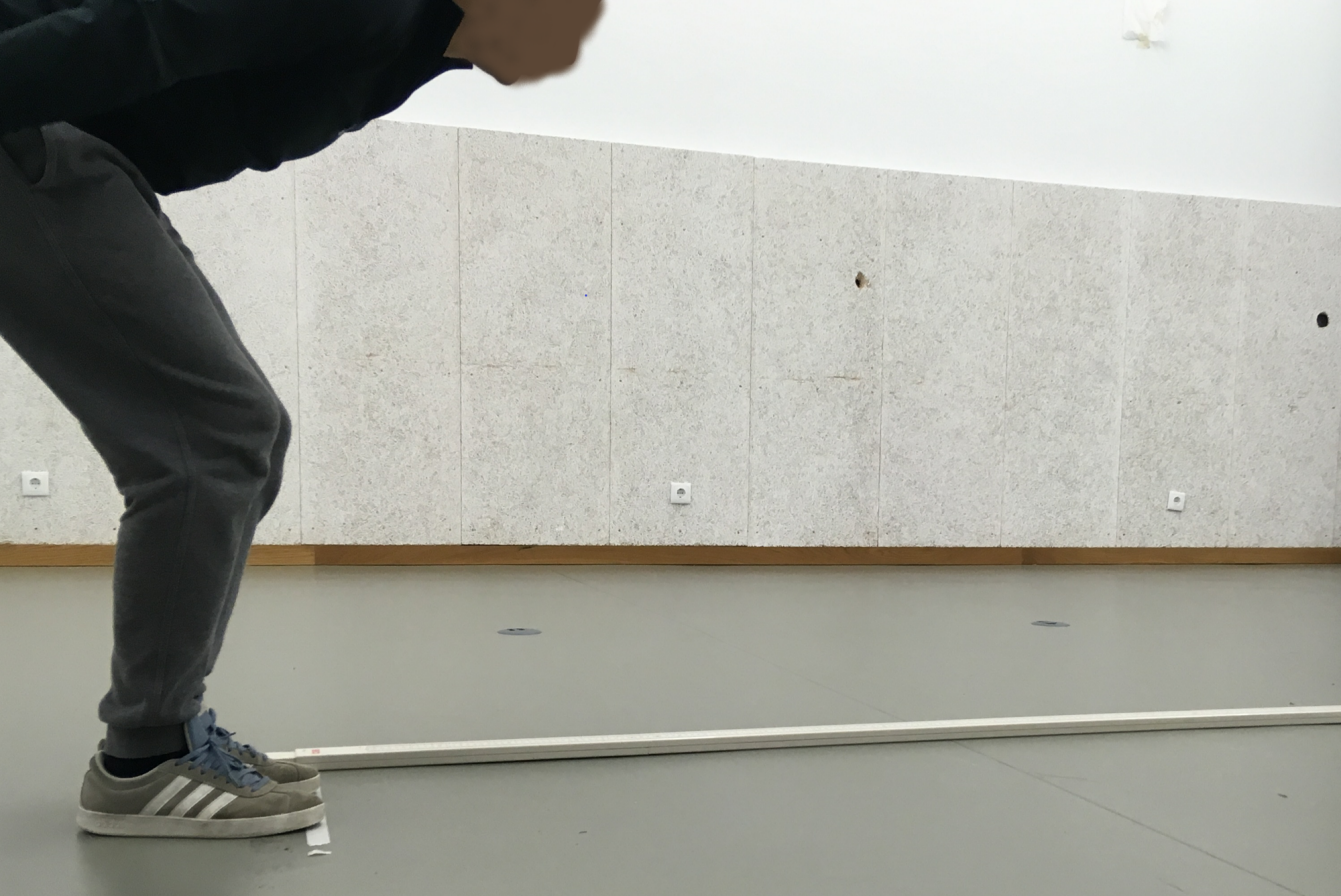
Motor competence—standing long jump test.

The **SLJ test** (Fig. 4) required participants to jump forward with both feet at the same time as far as possible. The test began with both of the participant’s feet placed behind the starting line. The longest distance between the starting line and the back of the heel at the landing spot after three attempts was scored (recorded in centimeters).

**Figure 5:**
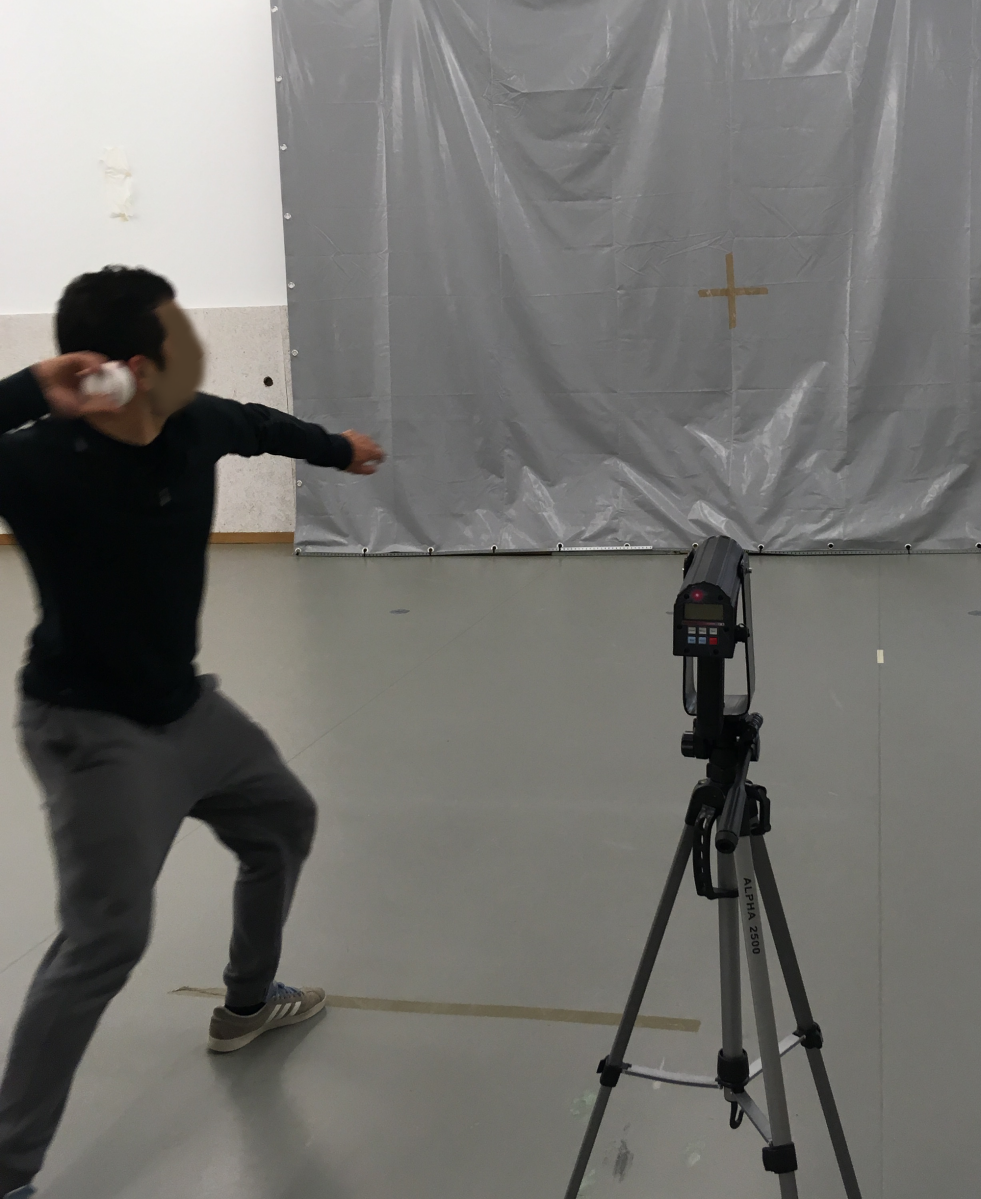
Motor competence—throwing velocity test.

**The** **TV test** (Fig. 5) required participants to throw a baseball (diameter: 7.3 cm; weight: 142 g) against a wall at maximum speed using an overarm action with a preparatory balance.

**Figure 6:**
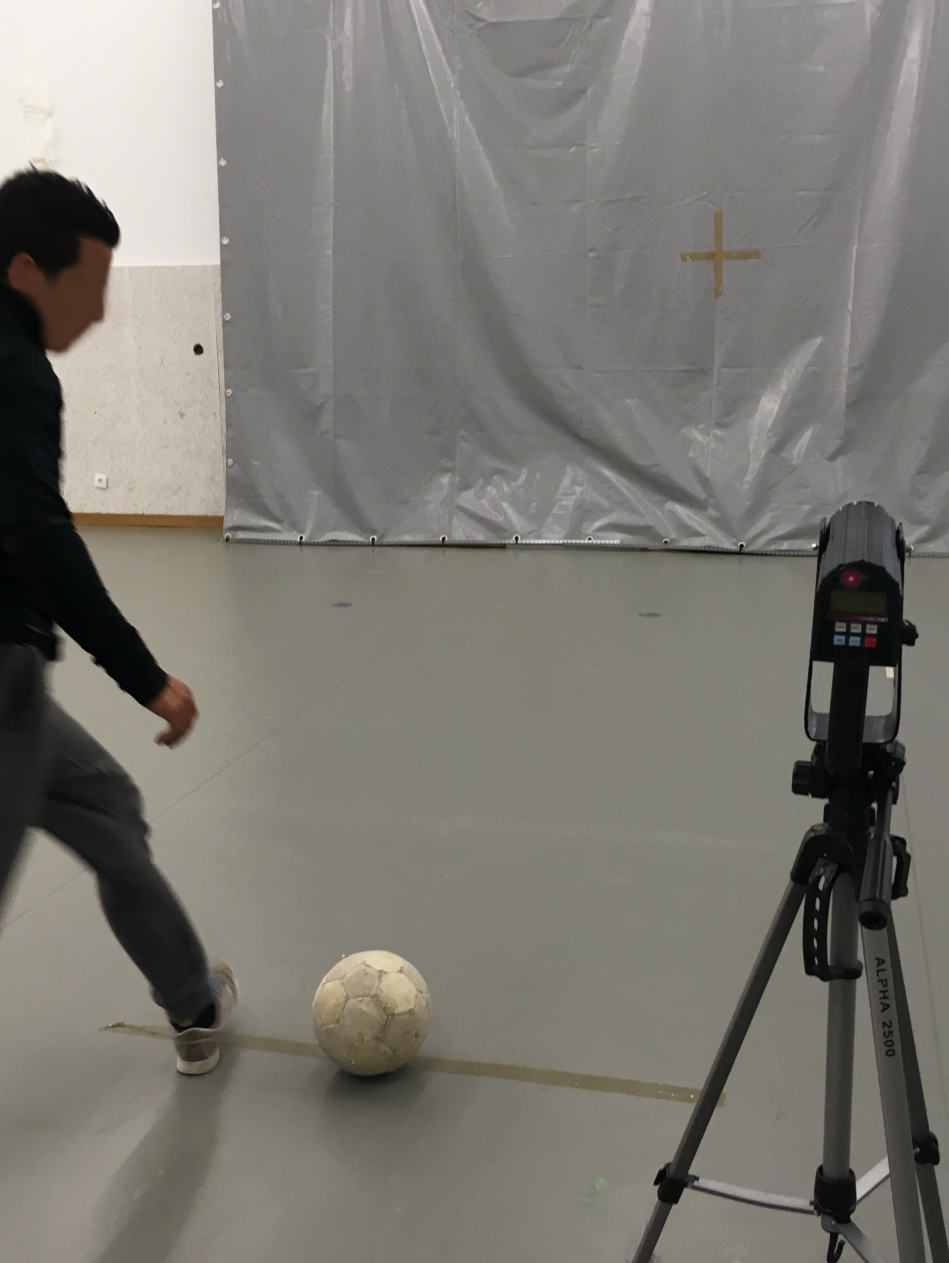
Motor competence—kicking velocity test.

The **KV test** (Fig. 6) required subjects to kick a size 5 soccer ball (circumference: 68 cm; weight: 410 g) against a wall at maximum speed using a preparatory balance.

The KV test and the TV test were performed with the participant’s preferred limb, and peak velocity was measured in m/s with a Stalker ATS II Radar System (Applied Concepts, Inc., Richardson, TX, USA). The radar gun was placed on a tripod and positioned behind a target marked on the wall in front of the kicking and throwing line. Each participant performed three trials; each participant’s best result was recorded.

Stability, locomotor, and manipulative category scores were calculated as the sum of the t-scores of the two tasks for each category. Inverse *t*-values were used for SHR, given that higher values represented lower performance. Total MC was calculated as the mean of the *t*-scores for all categories (Luz et al., 2016; Luz et al., 2017).

#### Statistical analyses

All test results were analyzed for the assumption of normality and the homoscedasticity of the tested sample groups. Descriptive statistics (average and 95% confidence interval (CI) for lower and upper limits) were calculated. The Mann–Whitney *U* test was used to compare males to females. The effect size (ES) for the non-parametric tests is obtained (Pallant, 2011): $r= \frac{ \left\vert z \right\vert }{\sqrt{N}} $, where *N* is the total sample size, and the value of *z* is reported after applying the Mann–Whitney *U* test. The classification of ES is obtained by using the following criteria (Pallant, 2011): very small effect (*r* < 0, 1); small effect (0, 1 ≤ *r* < 0, 3); medium effect (0, 3 ≤ *r* < 0, 5); and large effect (*r* ≥ 0, 5).

Spearman’s correlation test was conducted to examine the association between FMS™ scores and MCA results for all variables. The following correlation scale was adopted (Hopkins, Hopkins & Glass, 1996): trivial (*r* < 0.1), small (0.1 ≤ *r* < 0.3), moderate (0.3 ≤ *r* < 0.5), large (0.5 ≤ *r* < 0.7), very large (0.7 ≤ *r* < 0.9), nearly perfect (*r* ≥ 0.9). All statistical analyses were completed using SPSS software (version 22.0.0.0 for Windows, IBM, USA) for *p* < 0.05.

## Results

Anthropometric characteristics and comparisons between sexes are displayed in Table 1. This comparation demonstrate that male have statistically significant higher values for height, weight, bone mineral density and total lean mass while female demonstrate statistically significantly higher values for percentage of body fat (Table 1).

Functional Movement Screen and MC scores are presented in Table 2. The comparison analysis revealed that female had statistically significant better results in FMS^TM^ Active Straight Leg Raise than males. Meanwhile, males had significant statistically better results in FMS™ TSP and all MC components and constructs (Table 2).

**Table 2:** Functional Movement Screen and motor competence scores (mean and 95% CI).

**Variable**	**Men (*n* = 68)**	**Women (*n* = 24)**	***p*-value**	**Effect size**
FMS Deep Squat	2.0[1.8–2.2]	2.3 [2.0–2.5]	0.951	Very mall effect
FMS Hurdle Step	1.6 [1.4–1.8]	1.7 [1.3–2.0]	0.954	Very mall effect
FMS In Line Lunge	1.9 [1.7–2.0]	2.2 [1.8–2.5]	0.062	Small effect
FMS Shoulder Mobility	2.0 [1.6–2.3]	2.2 [1.8–2.6]	0.175	Small effect
FMS Active Straight Leg Raise	2.2 [2.0–2.5]	2.8 [2.6–3.0]^*^	0.008	Small effect
FMS Trunk Stability Push-Up	2.3 [2.0–2.6]^*^	1.2 [0.9–1.5]	0.000	Medium effect
FMS Rotary Stability	1.9 [1.9–2.0]	2.0 [2.0–2.0]	0.298	Small effect
FMS Composite Score	13.8 [12.9–14.6]	14.3 [13.4–15.2]	0.774	Very mall effect
MC Stability	99.4 [94.4–104.4]^*^	90.2 [84.3–96.1]	0.000	Medium effect
Shifting Platforms	31.3 [30.0–32.7]^*^	29.4 [28.1–30.6]	0.000	Medium effect
Lateral Jumps	49.9 [47.7–52.0]^*^	46.5 [44.2–48.8]	0.002	Medium effect
MC Manipulative	111.7 [108.6–114.8]^*^	75.7 [68.5–82.9]	0.000	Large effect
Throwing Velocity (m/s)	22.4 [21.6–23.3]^*^	15.2 [13.9–16.5]	0.000	Large effect
Kicking Velocity (m/s)	25.7 [25.0–26.4]^*^	17.8 [16.4–19.1]	0.000	Large effect
MC Locomotor	100.1 [95.2–105.0]^*^	100.0 [95.5–104.5]	0.004	Medium effect
Shuttle Run (s)	9.5 [8.8–10.1]^*^	11.0 [10.7–11.4]	0.000	Large effect
Standing Long Jump (cm)	221.6 [214.4–228.8]^*^	183.1 [167.6–198.6]	0.000	Large effect
Total MC	103.7 [100.9–106.5]^*^	88.6 [83.5–93.7]	0.000	Large effect

**Notes.**

FMS Functional Movement Screen cm centimeters m/s meters per seconds s seconds MC motor competence

* *p* < 0.05.

Table 3 shows the values for correlations between FMS™ scores and MCA components and constructs (locomotor (SHR and SLJ), stability (SP and LJ), and manipulative (TV and KV)). There are several magnitudes of correlation, with the majority being moderate (0.3 ≤ *r* < 0.5). The negative and significant correlations are more observed according to FMS™ ILL; FMS™ASLR and manipulative MC. All MC variables are significant correlated with FMS™ TSP (Table 3).

**Table 3:** Functional Movement Screen scores and motor competence constructs and tests scores correlations.

	**FMS deep squat**	**FMS hurdle step**	**FMS in line lunge**	**FMSshoulder mobility**	**FMS active straight leg raise**	**FMS trunk stability push-up**	**FMS rotary stability**	**FMScompositescore**
MC Stability	0.169	0.164	−0.009	0.237^*^	0.162	0.521^**^	−0.038	0.474^*^
Shifting Platforms	0.237^*^	0.184	−0.017	0.244^*^	0.147	0.372^**^	−0.010	0.363^**^
Lateral Jumps	0.038	0.184	−0.004	0.145	0.102	0.511^**^	−0.140	0.404^**^
MC Manipulative	−0.141	−0.084	−0.305^**^	−0.060	−0.203	0.474^**^	0.103	0.026
Throwing Velocity	−0.129	−0.105	−0.318^**^	−0.136	−0.210	0.440^**^	0.119	−0.017
Kicking Velocity	−0.124	−0.051	−0.249^*^	−0.028	−0.224^*^	0.433^**^	0.011	0.010
MC Locomotor	0.058	0.053	0.182	0.021	−0.080	0.294^**^	0.009	0.156
Shuttle Run	−0.019	−0.057	0.220	−0.131	0.153	−0.480^**^	−0.039	−0.198
Standing Long Jump	0.041	0.120	0.026	0.159	−0.052	0.462^**^	0.045	0.261^*^
Total MC Score	−0.027	0.027	−0.210	0.113	−0.050	0.521^**^	0.026	0.263^*^

**Notes.**

MC Motor Competence FMS Functional Movement Screen

* *p* < 0.05.

** *p* < 0.01.

When considering males and females separately, the analyses show that, for females (Table 4), there are statistically significant differences between: FMS™ DS and MC stability (*r* = 0.445; *p* = 0.033; positive and moderate); FMS™ TSP with MC manipulative (*r* = 0.563; *p* = 0.005; positive and large); SP (*r* = 0.456; *p* = 0.029; positive and moderate); LJ (*r* = 0.425; *p* = 0.013; positive and moderate); and SR (*r* =  − 0.476; *p* = 0.025; negative and moderate); FMS™ Composite Score and MC Stability (*r* = 0.454; *p* = 0.030; positive and moderate); and FMS™ Composite Score and Shifting Platforms (*r* = 0.556; *p* = 0.006; positive and large).

**Table 4:** Functional Movement Screen scores and motor competence constructs and tests scores correlations for female.

	**FMS deep squat**	**FMS hurdle step**	**FMS in line lunge**	**FMSshoulder mobility**	**FMS active straight leg raise**	**FMS trunk stability push-up**	**FMS rotary stability**	**FMScompositescore**
MC Stability	0.445^*^	−0.024	−0.068	0.371	0.373	0.563^**^	0.000	0.454^*^
Shifting Platforms	0.411	0.152	0.069	0.312	0.399	0.456^**^	0.000	0.509^*^
Lateral Jumps	0.289	−0.134	−0.185	0.247	0.098	0.425^*^	0.000	0.247
MC Manipulative	0.059	−0.067	0.007	−0.037	−0.30	−0.155	0.000	0.047
Throwing Velocity	−0.030	−0.040	−0.028	0.005	−0.246	−0.033	0.000	−0.012
Kicking Velocity	0.060	−0.161	0.079	−0.111	0.030	−0.239	0.000	−0.005
MC Locomotor	0.306	−0.155	0.411	−0.063	−0.111	−0.064	0.000	0.165
Shuttle Run	−0.090	−0.067	0.112	−0.289	0.094	−0.476^*^	0.000	−0.280
Standing Long Jump	0.292	0.113	0.259	0.102	0.090	0.343	0.000	0.334
Total MC Score	0.296	−0.165	0.005	0.072	0.043	0.078	0.000	0.205

**Notes.**

MC Motor Competence FMS Functional Movement Screen

* *p* < 0.05.

** *p* < 0.01.

There are 53% more statistically significant differences in males (Table 5) than in females. However, the magnitudes are similar but with different profile since the male have more statistically significant difference according to the FMS^TM^ composite score.

**Table 5:** Functional Movement Screen scores and motor competence constructs and tests scores correlations for male.

	**FMS deep squat**	**FMS hurdle step**	**FMS in line lunge**	**FMSShoulder mobility**	**FMS active straight leg raise**	**FMS trunk stability push-up**	**FMS rotary stability**	**FMScompositescore**
MC Stability	0.151	0.193	0.203	0.274	0.360^*^	0.301	0.008	0.510^**^
Shifting Platforms	0.278	0.142	0.098	0.302^*^	0.310^*^	0.064	0.071	0.303^*^
Lateral Jumps	−0.030	0.050	0.168	0.182	0.254	0.389^**^	−0.102	0.453^**^
MC Manipulative	−0.246	−0.187	−0.353^**^	0.053	0.007	0.098	0.294^*^	−0.053
Throwing Velocity	−0.212	−0.195	−0.367^**^	−0.050	0.047	0.061	0.313^*^ 0.016	−0.076
Kicking Velocity	−0.225	−0.073	−0.249	0.177	−0.005	0.075	0.161	−0.017
MC Locomotor	0.018	0.119	0.301^*^	0.140	0.063	0.119	0.091	0.144
Shuttle Run	−0.139	−0.112	0.006	−0.281^*^	−0.127	−0.097	−0.189	−0.242
Standing Long Jump	0.023	0.129	0.222	0.315^*^	0.147	0.144	0.170	0.248
Total MC Score	0.002	0.062	−0.019	0.276	0.353^*^	0.190	0.196	0.376^**^

**Notes.**

MC motor competence FMS Functional Movement Screen

* *p* < 0.05.

** *p* < 0.01.

## Discussion

The FMS™ does not explain fundamental movements based on motor control (locomotor, manipulative, and stabilizing tasks). Still, FMS™ Composite Score is positively and moderately associated with MC Stability construct and tasks; furthermore, FMS™ TSP explain 100% of the MC scores (Table 3).

Stability skills are related to non-locomotor acts, such as body rolling, bending, and twisting, the body, which characterize the ability to sense a shift in the interaction between body parts (balance), and the ability to adjust rapidly and appropriately to these changes (Gallahue, Ozmun & Goodway, 2012).

Essentially, the FMS™ is comprised of seven fundamental movement patterns that place the individual in extreme positions where weaknesses and imbalance become noticeable stability and mobility are not utilized appropriately (Cook et al., 2014b). Considering these concepts and the positive and moderate correlation between MC Stability and FMS™ Composite Score, is clear that the FMS™ predicts MC Stability (and vice versa) in young adults. However, the notion that the FMS™ patterns provide observable performance of basic locomotor and manipulative movements (Cook et al., 2014b) is not confirmed when assessed with product-oriented (quantitative) MC instruments. These notions are strengthened since the FMS™ ILL that focus on the stresses simulated during rotational, decelerating, and lateral type movements (Cook, Burton & Hoogenboom, 2014), presents a negative correlations with all MC manipulative (Table 3).

Each individual FMS™ test contributes to the final composite score and to specific clinical implications (Cook et al., 2014b). However, as demonstrated in other research, FMS™ TSP, when considered separately, can be reliable of physical function in specific populations (Silva, Clemente & Lourenco Martins, 2017). It can also be good indicator of balance when using measures of composite reach (Scudamore et al., 2018). Accordingly, this research also demonstrates statistically significant associations between FMS™ TSP and 100% of the MC stability, locomotor and manipulative tasks (Table 3). In young healthy adults, there are gender differences in muscle forces of the torso (Marras et al., 2001) that can mediate the results, since male and female demonstrate different correlation when analyzed separately (Tables 4 and 5). However, the MC shuttle run test presents a negative statically significant association with FMS™ TSP. This correlation was expected since the the 10 m shuttle run assesses speed and/or agility, (Ortega et al., 2008) and the FMS™ TSP require the trunk stabilizers to transfer force symmetrically from the upper extremities to the lower extremities and vice versa (Cook et al., 2014b). This factor may be mediated by the sample distribution (percentage of male and female) and the fact that female perform lower scores in the FMS™ TSP and MC tasks than men (Abraham, Sannasi & Nair, 2015; Bardenett et al., 2015; Luz et al., 2019; Luz et al., 2017; Silva et al., 2019). The observed statistically significant differences found between sexes, Table 1 (i.e., lower fat percentage and a higher BMD for males) are well-established in several studies (Alghadir, Gabr & Al-Eisa, 2015; Karastergiou et al., 2012). The same differences concerning higher MC and FMS™ scores for men (Table 2) are also reported in several works (Abraham, Sannasi & Nair, 2015; Bardenett et al., 2015; Luz et al., 2019). Considering the separate analyses, while still concerning the association between FMS™ scores and MC, very similar magnitudes and statistically significant correlations were observed between FMS^TM^ and MCand MC stability tasks, giving more strength to the notion that the FMS^TM^ predicts MC stability in young adults. However, as observed previously, this data may be mediated by the sample size of the present study and the fact that female perform differently than men (Abraham, Sannasi & Nair, 2015; Bardenett et al., 2015; Luz et al., 2019; Luz et al., 2017). In contrast, when compared with the results of the full sample and the female group, male all so presents significant correlation between FMS^TM^ ASLR, stability tasks and total MC (Table 5). These observations may be facilitated by the differences in the final score, when compared men and women (Table 2), and the fact that FMS^TM^ ASLR demands the demonstration of adequate hip mobility of the opposite leg and pelvic and core stability (Cook et al., 2014a). Indeed, the association between FMS^TM^ (qualitative) and MC (quantitative) measures is reinforced, as both composite scores also demonstrate significant associations in the male group. In the male group, the FMS™ ILL has a significant correlation with MC manipulative construct and tasks. The FMS™ ILL test challenges the trunk and extremities to resist rotation and maintain proper alignment (Cook et al., 2014b). A negative association in this variable was not expected, given that these manipulative tasks typically include a series of actions which involve grasping, moving and/or releasing an objects with the hands or feet (making these actions more challenging and complex than motor skills that don’t involve objects) (Gallahue, Ozmun & Goodway, 2012) and the joints of the hip, pelvis and spine need to aligned to perform many of the stabilizing functions that the body will require in order for the distal segment perform a specific function (eg. throwing) (Kibler, Press & Sciascia, 2006). This may affect the positive correlation between FMS™ RS and TV and MC Manipulative. Nevertheless, the positive association found in the present study is in accordance with the knowledge that FMS™ RS movement requires proper neuromuscular coordination and energy transfer from one segment of the body to another via the torso (Cook et al., 2014a).

In addition, like MC, FMS™, is influenced by the maturity status (becoming more precise after the mid-youth development phase) (Portas et al., 2016), excess weight (Silva et al., 2019), functional limitations, and motor skills (Duncan & Stanley, 2012).

The Functional Movement Screen is linked with MC Stability independent of being male or female.

These findings may be related to the fact that the FMS™ is a screening tool for individual movement inefficiencies and one’s readiness to return to physical activity after completing a rehabilitation program after suffering an injury or undergoing surgery (Cook et al., 2014b; Silva et al., 2017). Additionally, the FMS™ assesses functional mobility and postural stability without locomotion by using a set of tests that use external and internal rotation, hip flexion, core stability (Frost et al., 2012), which relate to MC Stability construct and tasks.

Besides the limitation inherent to the study design and sample type and size, it is important to remark that the quantitative method used to access MC can be observed as components of physical performance and not physical functioning as objectified by the FMS™. However, the MC battery used in the present study represents the three major latent variables of MC (i.e., stability, locomotor, and manipulative), all of which evaluated without a ceiling effect (Luz et al., 2016; Rodrigues et al., 2019). Since this is the first attempt to investigate this relationship, more research is needed to understand the nature of the link between the FMS™ and MC.

## Conclusions

In young adults, the FMS^TM^ explain fundamental movements based on motor control in the stability construct. Nevertheless, it was not established that FMS™ patterns provide observable performance of basic locomotor and manipulative movements.

The relationship between FMS™ scores and MC constructs are not clearly established in all domains. However, the FMS™ Trunk Stability Push is associated with better performance in all MC tasks and constructs.

The FMS™, on its own, is linked to objective measures of MC stability independent of being male or female.

##  Supplemental Information

10.7717/peerj.7270/supp-1 Supplemental Information 1 Association between motor competence and Functional Movement Screen Scores—dataset

## Additional Information and Declarations

### Data Availability

The following information was supplied regarding data availability: The raw data is available at Mendeley: Silva, Bruno (2019), “Functional Movement Screen and Motor Competence Assessment”, Mendeley Data, v1 10.17632/hcg2kzwbj2.1.
